# A framework for assessing the trustworthiness of scientific research findings^1^

**DOI:** 10.1073/pnas.2536736123

**Published:** 2026-02-03

**Authors:** Brian A. Nosek, David B. Allison, Kathleen Hall Jamieson, Marcia McNutt, A. Beau Nielsen, Susan M. Wolf

**Affiliations:** ^a^Center for Open Science, Charlottesville, VA 22911; ^b^Department of Psychology, University of Virginia, Charlottesville, VA 22904; ^c^School of Public Health, Department of Epidemiology and Biostatistics, Indiana University-Bloomington, Bloomington, IN 47405; ^d^Annenberg Public Policy Center, University of Pennsylvania, Philadelphia, PA 19104; ^e^National Academies of Sciences, Engineering, and Medicine, Washington, DC 20001; ^f^Law School, Medical School, and Consortium on Law and Values in Health, Environment and the Life Sciences, University of Minnesota, Minneapolis, MN 55455

**Keywords:** open science, metascience, research integrity, research ethics, assessment

## Abstract

Vigorous debate has erupted over the trustworthiness of scientific research findings in a number of domains. The question “what makes research findings trustworthy?” elicits different answers depending on whether the emphasis is on research integrity and ethics, research methods, transparency, inclusion, assessment and peer review, or scholarly communication. Each provides partial insight. We offer a systems approach that focuses on whether the research is accountable, evaluable, well-formulated, has been evaluated, controls for bias, reduces error, and whether the claims are warranted by the evidence. We tie each of these components to measurable indicators of trustworthiness for evaluating the research itself, the researchers conducting the research, and the organizations supporting the research. Our goals are to offer a framework that can be applied across methods, approaches, and disciplines and to foster innovation in development of trustworthiness indicators. Developing valid indicators will improve the conduct and assessment of research and, ultimately, public understanding and trust.

The purpose of scientific research is to generate generalizable knowledge. Many factors can interfere with that pursuit. Developing hypotheses, designing tests, rooting out errors, and exploring the unknown require creativity, rigor, and persistence in the face of ambiguity, false starts, and dead ends. Because scientific inquiry exists within a culture of critique and correction, claims and evidence produced by some researchers are challenged by others.

Making progress involves intellectual humility, including awareness that there are known and unknown uncertainties.

Knowledge production is a hard, slow process. It is even harder and slower when research findings are not trustworthy. In this article, we present a *Trustworthiness Framework for Assessing Research Findings* for the research community, identify opportunities to create and improve indicators of research trustworthiness, and discuss how such indicators can improve research culture and practice in pursuit of knowledge. The audiences for this paper include researchers, individuals, and communities participating in research, leaders, and policymakers who shape research assessment and reward systems, peer reviewers and journal editors, and journalists communicating to the public about research.

By research findings, we mean the evidence and claims produced in the research process. Trustworthy is not synonymous with correct or true. Trustworthy research findings are those that contribute to the social processes of knowledge production. Beyond our scope are questions of how research participants and collaborating communities assess the trustworthiness of researchers or research organizations. Also beyond our scope is describing how trustworthy research findings are used to develop trustworthy models and theories.

Scientific research is conducted using a wide range of methods, with a variety of epistemologies, at scales from modest to vast, in many fields, and on countless topics. Assessing the trustworthiness of findings differs across these contexts. This paper works from the premise that identification of shared principles and themes can provide common language and improve understanding across research domains. Simultaneously, pursuing a common framework can help clarify when some concepts do not apply across all research domains.

## The Trustworthiness Framework for Assessing Research Findings

We derived the *Trustworthiness Framework for Assessing Research Findings* framework from the literature on scientific rigor and validity, reproducibility and replicability, public indicators of scientific trustworthiness, and how trustworthiness can fail. There are seven distinct components that contribute to trustworthiness of research findings including whether the research is accountable, is evaluable, has been evaluated, is well formulated, controls bias, reduces error, and whether the claims are warranted by the evidence. Those components are relevant at three levels that contribute to trustworthiness: the research itself, researchers conducting and evaluating the research, and organizations–including institutions, funders, and journals–facilitating and supporting the research. A visual representation of the framework is presented as *SI Appendix*, Table S1. To ensure broad applicability across scientific domains, the framework has the following features:

• Systems level perspective: Establishing trustworthiness of research findings is not exclusively a function of the findings themselves, but is also a function of the actions by researchers to produce them, and the context and support systems in place at research supporting organizations. For example, the extent to which research findings have been evaluated depends upon factors such as peer review of research outputs, participation by the researcher in scholarly and public discourse about their program of research, and adherence to rigorous research assessment practices by organizations.
• Behavioral: The trustworthiness of findings is earned primarily through actions in designing investigations, producing credible evidence, making sensible claims from that evidence, transparently sharing the research for examination by others, and providing oversight and support of that work. The framework focuses on behaviors and actions that are direct indicators of trustworthiness and not proxy indicators such as reputation. For example, accountability is advanced when researchers properly credit contributors and disclose conflicts and funders, when Institutional Review Boards (IRBs) and other oversight bodies exercise due diligence, and when universities reward trustworthy research.
• Measurable: The framework prioritizes indicators that can, in principle, be measured to assess research trustworthiness. Some indicators are readily measurable (e.g., sample size, statistical power, and reliability). For others, innovation in measurement is needed, such as offering indicators of the extent to which research is well calibrated, with claims that are warranted by the evidence.
• Inclusive: The framework prioritizes components and indicators that have broad applicability across a range of scientific research activities, including both quantitative and qualitative research. For example, Lincoln and Guba’s criteria for trustworthiness of qualitative research can be mapped to the framework’s components: evaluated for dependability and confirmability, well-formulated for transferability, and well-calibrated, controlling bias, and reducing error for credibility (1). Lincoln and Guba articulate more concretely how those components are operationalized in qualitative work (1).
• Improvable: The examples of indicators are not exhaustive. The framework is designed to stimulate further research and improvements in the components, levels of analysis, and indicators assessing trustworthiness.

This *Trustworthiness Framework for Assessing Research Findings* integrates trustworthiness perspectives across domains that include: research integrity and ethics, research methods, research transparency and openness, research inclusivity, research assessment and peer review, and research communication.

## Components of the Trustworthiness Framework for Assessing Research Findings

In this section, we briefly justify each component of trustworthiness and offer examples of indicators that can be assessed.

### Accountable.

Are the researchers accountable for conducting research in a trustworthy way? Researchers who are accountable and ethical engage in behaviors and follow practices that enhance the trustworthiness of research findings. Accountability begins with institutional review of research by bodies such as an IRB, Institutional Animal Care and Use Committee (IACUC), and a Data Safety and Monitoring Board to ensure that research adheres to ethical standards and safeguards rights and well-being (2–5). For example, in U.S. medical research covered by the Common Rule or FDA equivalent (or conducted by researchers in institutions “checking the box” to offer broad Federalwide Assurance that all research will comply with the federal regulations), IRB approval is essential before conducting clinical trials to protect the rights and safety of participants. Researchers and trainees take steps to enhance the trustworthiness of their findings through education and training in rigorous and ethical research practice. Research institutions contribute to trustworthiness of findings by offering needed training and mentorship, and by supporting competent IRBs and IACUCs that oversee institutional research. Poor operation of institutional accountability safeguards threatens the trustworthiness of research findings (6, 7).

Researchers, who have a general interest in producing findings that are career advancing, sometimes have conflicts of interest (COI) or conflicts of commitment that could influence the research process or findings. Disclosure of financial interests and personal affiliations helps research institutions monitor and manage such conflicts, and helps journals and readers assess sources of possible bias (8, 9). For example, pharmaceutical research conducted by researchers with a financial interest in the drug creates the conditions for motivated reasoning to report findings that indicate effectiveness and ignore findings that do not. Disclosure may not be sufficient to eliminate or even manage the potential influence of such conflicts, but it invites additional scrutiny of the research process for potential bias and allows management of the conflict (10). Researchers contribute to trustworthiness of findings produced in the research by completing disclosure statements regularly to identify financial interests that could affect choices of research topics, methods, and reporting. And institutions bolster perception of the trustworthiness of research findings by articulating a COI policy and having effective procedures to manage and mitigate potential conflicts.

Disclosure of funding sources acknowledges both potential conflicts of interest for the researchers, and the potential influence of the funding organization on the study design, data collection, analysis, and reporting of the research findings (11). Transparency of funding sources helps potential participants and readers understand potential influences on the research. It also may increase trustworthiness by discouraging funder influence on research and by making potential sources of bias visible for examination. In nutrition research, for example, industry-funded studies may be less likely to examine dietary behaviors than non-industry-funded studies (12). The National Academies report on genetically engineered crops avoided using research funded by organizations that had a financial or ideological stake in the outcomes (13).

In qualitative research, positionality statements are a way of acknowledging researchers’ subjectivity and potential biases. In such disclosures, researchers state their perspectives, experiences, and cultural backgrounds, as these may shape their approach to the research process and interpretation of findings (14). Positionality statements offer a broader conception than conflicts of interest of how researchers’ own perspectives may shape their research findings. There is a lack of evidence on whether such statements are effective in reducing bias in research findings and on how they are used in interpretation of findings (15). They are, nevertheless, an effort to make potential biases more transparent.

Proper acknowledgment of individuals who contributed to the study design, data collection, analysis, and interpretation ensures that all contributors receive appropriate recognition for their work, and are accountable for it (16, 17). Historically, disciplinary norms about the meaning of the author order were considered sufficient for acknowledgement of contributions. But, concerns about gift or honorary authorship, ghost authorship, large-scale collaborations, and inequitable practices in assigning credit have motivated greater specificity in reporting research contributions (18). The CRediT taxonomy makes it possible for authors to specify their contributions to increase transparency and accountability for production of research findings (19).

Research organizations such as universities play an important role in establishing a research culture that holds researchers accountable and incentivizes trustworthy research. The primary accountability mechanisms are training, research oversight, compliance systems, and required reporting of potential conflicts. Key incentives in research institutions are hiring, promotion, and tenure. If those reward systems favor publishing mostly positive, novel findings regardless of whether research practices are transparent and rigorous, then researchers will adapt to these measures of success (20). In such circumstances, researchers who continue to prioritize rigor and transparency may be less likely to advance in a competitive process for career advancement (21). By offering a reward system that holds researchers accountable for trustworthy research practices to advance and keep their job, institutions increase the likelihood that their researchers will produce trustworthy research findings.

### Evaluable.

Can the research be assessed? Research is a show-me enterprise. Transparency and sharing enable others to determine the credibility of the claims based on the evidence (22, 23). Transparency of research process and content is another form of accountability for researchers, in addition to the emphasis on ethical practices noted in the prior section. The open science movement has expanded recognition that transparency and sharing mean more than making the paper available, which Buckheit and Donoho dismiss as inadequate (24). Open science also refers to sharing research methods and findings including data, materials, and code, and the plans, protocols, and processes involved in conducting the research. Sharing the content of the research enables others to evaluate reproducibility or reuse and adapt the content to assess reliability or validity. And, sharing the process of research makes it possible to assess whether behaviors occurred that could enhance or detract from the trustworthiness of the findings. For example, were steps taken to reduce questionable research practices (25, 26) and avoid p-hacking that could inflate effect sizes and the likelihood of observing false positives (27)? Moreover, failure to report research processes and outcomes impedes the ability to assess the research. This is particularly true when publication ignores negative and null results (28, 29). Transparency of research facilitates assessment of the individual study, the accumulating evidence, and appropriate next steps in the field.

Beyond transparency and sharing the content and process of findings, researchers foster the trustworthiness of their research through public engagement on research approaches and public representation of their work and research practices. Research institutions and funders promote evaluability of research findings by adopting policies like the Transparency and Openness Promotion Guidelines outlining the expectations for research supported by the institution (22), by ensuring open access to research findings to foster public scrutiny, and by increasing the transparency of the processes that determine which research is funded.

### Evaluated.

Has the research been assessed? Subjecting the research to critique and correction is critical for establishing its trustworthiness. A basic operating assumption of research as a social activity is that research findings are critically evaluated by others who are independent of the researchers who produced them. This systemic, social activity of peer review aims to root out bias, identify alternative explanations for evidence, and ultimately advance knowledge (30).

Individual researchers improve the trustworthiness of research findings by subjecting them to scrutiny in academic conferences, journals, and other scholarly forums, and by the communities that are interested in or affected by the work. The social component of scholarly research can be underappreciated. Open discussion and debate allow researchers to address potential weaknesses, explore alternative explanations, and refine their interpretations. By participating in such exchanges, researchers subject their work to a wider community of experts, leading to greater scrutiny and, ultimately, more reliable findings.

Part of research evaluation is critical assessment of research findings with reason and logic. Another part is conducting more research to test or bolster prior research findings. Rigorous assessment may identify a possible alternative explanation for a finding. Follow-on research may distinguish the viability of the original versus alternative explanations, assess replicability by testing the same question with new data, assess robustness by testing the same question using different approaches to analyzing and interpreting the original data, or assess reproducibility by redoing the original analysis with the original data (31, 32).

Organizations such as funders, universities, and private companies conducting research improve the trustworthiness of research findings by creating the systems and reward structures for the evaluation of research, and addressing biases in evaluation systems (33). For example, research assessment processes conducted by funding agencies, academic institutions, and expert panels evaluate methodological rigor, significance, and the level of innovation. Rigorous research assessments provide an external validation of research findings and inform decisions related to funding and recognition. In the evaluation of grant proposals, for instance, funding agencies prioritize projects that have the potential to make meaningful contributions to the field and are based on sound methodologies.

Journals and publishers can also create evaluation processes to improve research trustworthiness. For example, the publishing model, Registered Reports, conducts evaluation prior to knowing the research outcomes so that the decision to publish is focused on the formulation of the question and quality of the methods, rather than the novelty of the observed outcomes (34, 35). Observational evidence suggests that shifting the primary evaluation away from the findings is associated with greater rigor and quality of research (36), and with a lower likelihood of ignoring negative or null findings (37). In contentious areas, precommitment via preregistration or Registered Reports promotes trustworthiness by establishing agreement on research designs and articulation of opposing predictions prior to knowing the outcomes of the research (38, 39).

Research assessment by institutions directly shapes the behavior of researchers producing findings by setting the expectations and criteria by which their work is evaluated. Recent efforts such as the Higher Education Leadership Initiative for Open Scholarship (HELIOS Open) and Coalition for Advancing Research Assessment (CoARA) have identified the criteria for research assessment as a critical priority for reform so that researchers are evaluated using criteria aligned with trustworthy research (40).

### Well-Formulated.

Does the research take into account relevant knowledge and perspectives? Contribution to knowledge production is more effective when new research takes current knowledge and evidence into account. Conducting a thorough review of the research literature and engaging with diverse perspectives and affected communities clarifies how new research can contribute to knowledge production by addressing existing gaps, introducing new possibilities, or providing confirmatory or contradictory evidence to existing understanding. For example, in a study investigating the impact of a new teaching method on student learning outcomes, a literature review would help researchers identify similar interventions and compare their results, thereby refining the research approach and connecting the findings with other relevant evidence. Researchers can increase the trustworthiness of their findings by considering new, alternative, and opposing perspectives to better anticipate potential objections to their approach and interpretation, and to help them design research that is more likely to yield new insights.

Generating hypotheses that are informed by existing theory and evidence ensures that research questions will genuinely contribute to generalizable knowledge. By explicitly grounding hypotheses in well-defined theories, researchers can better interpret results and propose more coherent explanations for observed outcomes. Conversely, research that challenges existing theories is more compelling when it clarifies how the new research challenges their formulation. For example, in psychological research on memory retention, hypotheses based on cognitive theories of memory are most likely to yield insights that advance the field and shed light on the validity of the underlying theories. And, if the research proposes an alternative theoretical perspective, the strongest evidence may come from research designs for which the opposing theoretical perspectives make different predictions (41).

Another aspect of conducting well-formulated research is matching the research design with the population of interest so that the research findings are applicable. Researchers can promote the external validity of their findings by defining representativeness in the context of their research and using methods, such as random sampling, to achieve it. For example, in neuroscience and preclinical research, the emerging evidence of influence of biological sex of animals on research findings calls into question prior research that ignored this variable (42, 43). Likewise, lack of representativeness in public health, social, or behavioral research might miss important sources of variability across the population that constrain the applicability and interpretation of research findings (44–47).

Research institutions foster well-formulated research by creating conditions that include a variety of stakeholders in the research process (48, 49). For example, for research in public health, funders can support participatory research with communities to help researchers formulate the research questions and best methods to use. Research institutions can promote research that considers a diversity of views and perspectives by hiring a diverse research staff, fostering robust discussion, and promoting a scholarly culture that is productively skeptical. Finally, research institutions can provide communication mechanisms that make their research plans, methods, and findings more accessible and understandable to all stakeholders thereby promoting engagement in the research process and effective translation of research findings into practice.

### Controls Bias.

Does the research promote accuracy and validity? When aiming at a target, missing the target consistently to the left or right is evidence of bias. Something in the process is producing systematic error. Rigorous practices identify and mitigate biases that lead researchers to incorrect claims. Such biases may emerge as a consequence of the research design, the context of conducting the research, or the actions of researchers, whether intentional or unintentional.

Validity frameworks identify how research methods can address biases that lead to inaccuracy. For example, internal validity refers to the extent to which the observed findings support a relationship between cause and effect (50). There are a variety of methods to eliminate alternative explanations such as randomization of subjects to experimental conditions to eliminate confounding influences on the observed relationship. And, for research topics for which randomization is infeasible or impossible, there are ways to mitigate confounding influences when making causal inferences (51–53). Blinding researchers from the condition assignments or other features during data collection or data analysis reduces the experimenter biases that can unintentionally alter outcomes (54–56). The need for blinding is illustrated by a classic demonstration in which research assistants recorded the length of time that rats required to learn a maze. Those who were randomly told that their rat was smart recorded faster maze-learning than those randomly informed that their rat was dumb (57).

A complement to blinding is preregistration of a research design and analysis plan prior to observing the study outcomes. This can protect against confirmation bias, hindsight bias, and outcome bias (58, 59). For example, if a study fails to support the researcher’s hypothesis, the researcher might rationalize the failure as being due to faulty methods and ignore the evidence. Preregistration clarifies whether analysis decisions were planned in advance or made after the fact, making potential bias more evident. Also, registration ensures that the study is discoverable, regardless of whether it is ultimately published.

Construct validity refers to the extent to which the study measurements assess the constructs or concepts of interest (50). For example, a researcher investigating the influence of feeling proud on charitable giving might try to experimentally create the experience of pride, but inadvertently also create feelings of happiness and surprise. This would create difficulty in isolating the causal influence of pride compared with other emotions. Alternatively, if a test is intended to measure intelligence, but performance is influenced by cultural references (such as assuming that the test taker knows the rules of cricket to answer a math question), the measure would provide misleading results. Construct and test validation contribute to the trustworthiness of research findings.

Researchers increase trustworthiness of their findings by being knowledgeable about the strengths and limitations of existing and emerging methods for conducting research on their topic. Keeping up with these innovations with training and retraining is a positive indicator of pursuing trustworthy research findings.

Research organizations promote accuracy and validity in the production of research findings by supporting mechanisms for pursuing rigorous research, particularly instrumentation and services for advancing validity. For example, many research-intensive medical centers operate core facilities for conducting research based on methods and techniques that are widely used across the institution. At their best, such facilities localize expertise and competent execution for maximizing validity. This can include standard operating procedures for validating antibodies or other activities that ensure appropriate use and valid outcomes. Likewise, institutions invest in other types of instrumentation that is shared across many researchers and teams that might otherwise be inaccessible to any individual or group. For example, leading-edge neuroimaging machines for neuroscience research and telescopes for astronomy may be out of reach for individual laboratories, but become accessible with institutional investment.

### Reduces Error.

Does the research promote precision and reliability? When aiming at a target, the unsystematic dispersal of attempts to hit the target is an error in reliability and precision.

Something in the process is producing errors that thwart hitting the target reliably and consistently. Virtually all areas of research wrestle with separating signal from noise to determine whether a research finding is due to a regularity in the world or to happenstance. Improving precision and reliability improves trustworthiness of research by reducing the likelihood that research findings are due to mistaking noise for signal.

Precision refers to the degree of exactness with which measurements are made, and reliability refers to consistency of observations across repeated measurements. Researchers can improve precision and reliability by employing sensitive and standardized measurement tools, calibrating instruments properly, and carefully controlling extraneous variables. For example, continuous improvement in telescope sensitivity has enabled more precise measurement of more distant celestial objects (60), and continuous improvement in scales measuring weight has increased the consistency of outcomes across repeated measurements (61).

Another way of improving reliability is having a large enough sample size to confidently distinguish signal from noise. Depending on the research application, sample size may refer to repeated measurements of the same thing, observation of many things, or both. A widespread problem in research is a study sample size too small to reliably detect the phenomenon of interest (62–65). This leads to false positives and lower trustworthiness of findings (63). This can be addressed by conducting power analyses to estimate the sample size needed based on expectations of the likely magnitude of the effect of interest (66), or by planning sample size based on the smallest effect of interest (67). In qualitative research, there are complementary concepts of saturation and information power to determine whether the sample size is sufficient (68–70).

Researchers advance precision and reliability when they ensure they have adequate resources to investigate the scientific questions of concern. For example, a common challenge in some fields is that testing hypotheses of interactions between multiple variables requires substantially larger sample sizes than are usually available, leading to underpowered tests and false discoveries (71, 72). When researchers need to use measurement tools whose precision and reliability are not optimized, then their options for productive investigation are to investigate phenomena that elicit large effect sizes, or to gather massive amounts of data to reliably extract signal from substantial noise. Researchers who properly calibrate their research questions to their available resources and tools will produce more trustworthy findings.

Research organizations promote precision and reliability by supporting the accumulation of evidence across multiple investigations. This can include supporting data repositories and study registries for researchers to share data. It also can include offering rewards for aggregating evidence. A fieldwide institutional investment in promoting precision and reliability is the Cochrane collaboration that synthesizes evidence in medical research to offer trustworthy findings for health professionals, patients, and policymakers (73). This effort reflects the understanding that less precision can be tolerated in individual investigations if there is investment in aggregating evidence for precise conclusions when translating findings into practice. Finally, research organizations promote precision and reliability by investing in infrastructure and providing adequate funding for research to ensure that real progress can be made.

### Well-Calibrated.

Are the claims warranted by the evidence? Theories, models, and explanations of the world are approximations and simplifications based on the best available evidence. Research progress is marked by identifying the limitations of present understanding and offering new explanations that better describe, predict, and explain reality. This means that all research claims supporting theories and models need to be rigorously evaluated.

All research findings are subject to interpretation. Scientific claims almost always exceed the study’s evidence because they are intended to be about potential regularities in the world. There are always uncertainties, constraints on generality, and alternative explanations for what was observed. As noted in the National Academies report on reproducibility and replicability (31), “Researchers should, as applicable to the specific study, provide an accurate and appropriate characterization of relevant uncertainties when they report or publish their research. Researchers should thoughtfully communicate all recognized uncertainties and estimate or acknowledge other potential sources of uncertainty that bear on their results, including stochastic uncertainties and uncertainties in measurement, computation, knowledge, modeling, and methods of analysis.” When interpreting their results and making claims, researchers should identify these limitations transparently, make clear their impact on the interpretation of their results, and discuss how further research could address them.

Researchers promote trustworthiness of their findings by interpreting results carefully and cultivating an openness to seeking counterevidence for their claims. This is a necessary part of the research, interpretation, and publication process. Participation in scholarly presentations and debate including posting a preprint for feedback can help expose their claims and evidence to skeptical inquiry by others, contributing to the trustworthiness of their research.

Research organizations promote trustworthiness by avoiding rewards for exaggerated claims. Research assessment that focuses on the quality and rigor of methods may discourage exaggeration and misleading spin, and reduce pressure on researchers to produce exciting claims instead of reliable evidence. Organizations also can create disincentives for spin in communicating research to the public, and ensure the organization places higher value on gaining a reputation for scientific excellence rather than mere novelty. Finally, publishers and research organizations can incentivize researcher correction of errors by applauding rather than penalizing scholars who identify and promptly correct them and by distinguishing voluntary from involuntary correction or withdrawal of published work (74, 75).

## Avoiding Overreliance on Proxy Indicators of Trustworthiness of Research Findings

The framework and review of indicators provides some insight on how research findings become trustworthy. Of critical interest is how can a consumer of research know whether the framework’s criteria have been met for any given research finding? For example, none of the articulated criteria relies on institution prestige, researcher fame, or the desirability of a finding as a basis for its trustworthiness. Yet these are widespread influences on presumed trustworthiness. In the present research culture, there is too little emphasis on providing evidence for the framework’s criteria. Instead, there is an overreliance on proxy indicators deemed to imply that some of these criteria have been met.

Scholarly research has leaned on peer review followed by publication as a proxy indicator for trustworthiness of research findings. The proxy indicator presumes that if findings are published in a peer-reviewed journal, then the reader can be confident that independent researchers examined them on a variety of dimensions and confirmed the evidence and claims. Moreover, if the journal has a strong reputation, then the credibility of papers and claims in that journal is enhanced.

There are several problems of overreliance on “published in a peer-reviewed journal” versus “not published” to represent the trustworthiness of research findings. Trustworthiness of findings is more complex than this indicator can represent. Peer review is not designed for, or capable of, providing a comprehensive assessment of the trustworthiness of research findings. Indeed, the limited reliability and validity of peer review is well known (33, 76–78), and the rigor of peer review varies substantially from journal to journal. The stature of a journal is also a highly imperfect proxy. Journal Impact Factor, an estimate of average citation frequency of papers in a journal, is sometimes errantly used as an indicator of trustworthiness. By this metric, papers in higher impact journals are more trustworthy than others. However, there is little support for that claim (79–81).

An even more daunting challenge is the academic reward system in which the number of publications in peer-reviewed journals is the currency of advancement. When “published in a peer reviewed journal” becomes a proxy for trustworthiness, high numbers of peer-reviewed publications can offer the veneer of trustworthiness replacing the need to conduct genuinely trustworthy research. Predatory journals publish articles with little to no quality control. Paper mills add authors to papers for a fee. These dysfunctional markets create the illusion of integrity by leaning on the ease of mimicking proxy indicators. In sum, “published” or “peer reviewed” can encompass low validity, low reliability research published by individuals or publishers that are gaming the system. Alternative, direct indicators are needed.

## Toward More and Better Indicators of Trustworthiness of Research Findings

The *Trustworthiness Framework for Assessing Research Findings* presupposes that trustworthiness is complex and multifaceted. Achieving research credibility involves many actions by researchers and research-supporting institutions. No single one determines whether a finding is trustworthy. But, knowing what actions were taken, and the quality of those actions, can support the scholarly discussion about the trustworthiness of research findings and their use.

Table 1 provides examples of ways to assess the quality, usefulness, and generalizability of trustworthiness indicators like those presented above. *SI Appendix*, Table S2 provides a longer list. In practice, there is no perfect indicator, and development of indicators often involves tradeoffs. For example, it is tempting to use an indicator that is easy to measure but not reliable or valid rather than a reliable and valid one that is difficult to measure. Innovation can reduce or eliminate those trade-offs by, for example, dramatically reducing the difficulty of measuring a valid indicator that was previously impractical to use.

**Table 1.** Potential indicators of the trustworthiness of research findings can be assessed in a variety of ways to determine their quality, usefulness, and generalizability

Assessment Criterion	Explanation	Example A	Example B
Done versus done well	The indicator measures whether the action was performed or not versus the indicator assesses the quality of performing the action.	Authors reporting that they randomized the experiment reflects whether the action was performed, not the quality of randomization.	An assessment including whether the dataset is findable, accessible, interoperable, and reusable (FAIR) is determining whether the indicator, shared data, is done well.
Self-certification versus independent verification	Performance on the indicator is assessed by the actor(s) themselves versus by an independent source.	Authors reporting that they blinded the experiment condition is self-certifying by the authors that they did it.	An assessment of whether another research group could replicate a finding reflects independent verification of an indicator of replicability.
Human versus automated	The indicator is based on human judgment versus the result of an automated process.	Peer assessments, such as judgment of effectiveness of assessing stakeholder interests in the research, is based on human judgment.	Machine learning extraction of evidence for use of reporting guidelines in papers is an automated indicator.
Thin versus thick	Assessment of the indicator draws on a narrow representation of the construct of interest versus a broad representation.	Measuring accountability of the research as exclusively disclosure of conflicts of interest is a thin assessment of accountability.	The indicator, received tenure at an academic institution, is usually the product of a thick assessment of the researchers’ scholarly record.
Domain-specific versus domain-general	The indicator is applicable to a specific method or topic versus applicable to a wide range of methods and topics.	The indicator, a priori power analysis performed, is specific to methodologies for which power analysis is relevant.	The indicator, peer assessment of researcher engagement in scholarly discourse, can be applied across methods and topics.
Open versus proprietary	The assessment is openly available for use and reuse versus owned or controlled by a specific entity.	An openly licensed rubric for evaluating presence of confounds can be used and reused by anyone.	Journal impact factor, as implemented by Clarivate Analytics, is a proprietary measure.

Six examples are presented here, 14 are presented in *SI Appendix*.

### Innovations in directly evaluating research findings.

Some journals have taken the straightforward but important step of making peer reviews of published papers publicly accessible. This action provides transparency of the evaluation process and is an incremental step away from publication as a dichotomous assessment. Likewise, many journals have implemented policies requiring disclosure of conflicts and funding sources, sharing of data and code, and use of data reporting standards—all to increase the accountability and evaluability of research findings.

Entrepreneurial groups are testing innovations in peer review and evaluation practices. For example, RepliCATS provides a structured, collaborative review process to assess research (82). The Social Science Prediction Platform evaluates research predictions before the results are known (83). The Institute for Replication conducts reproductions of published findings to verify that the reported results are reproducible from shared data and code (84). And, the ERROR service incentivizes reviewers with bounties to find errors in published articles (85). These novel methods of leveraging and surfacing peer assessments are expanding the insight and reach of factors that affect the trustworthiness of findings.

The AI and machine learning revolution also is affecting research assessment. For example, the Dataseer.ai commercial service offers AI methods to scan research papers to extract open science indicators such as open access publishing, preprints, open data, citation of data reuse, and open code. These indicators are provided as dashboards to institutions and other stakeholders to monitor research activities that make research more evaluable. Similarly, SciScore automatically extracts insights about transparency of research papers using indicators derived from reporting standards such as MDAR (86) and ARRIVE (87).

Several research groups have developed machine-based methods to assess the quality or anticipated replicability of research findings, with accuracy rates that rival human judgments (88–91). Relatedly, services such as StatCheck automatically review papers for statistical reporting errors (92). If valid and generalizable, such machine-based solutions could dramatically increase the scalability of indicators that assess specific qualities of research findings.

### Innovations in evaluating researchers.

The Coalition for Advancing Research Assessment (CoARA) (93) and Higher Education Leadership Initiative for Open Scholarship (94) initiatives are promoting evolution in researcher assessment toward criteria related to research integrity and trustworthiness and away from criteria that incentivize dysfunctional research practices. For example, research institutions that sign-on to CoARA commit to reviewing and updating their researcher evaluation standards by recognizing there are many ways to contribute to research, that many forms of research assessment should be qualitative and rooted in peer assessment, and that research rankings can be dysfunctional for promoting trustworthy research. Signing organizations commit to completing reform of their assessment standards within 3 y. Each institution is responsible for determining its own indicators, but working groups and information sharing promote broad engagement and identification of good practices.

### Innovations in evaluating research institutions.

The UK Committee on Research Integrity has offered 16 indicators for higher-education institutions to promote research integrity in leadership, strategy, procedures, practices, and skills (95). The indicators include publicly sharing an institutional strategy for promoting integrity, conducting internal audits or reviews for compliance monitoring, and assessing adoption of training and training effectiveness.

The Enhancing Quality in Preclinical Data consortium has developed a quality system for preclinical research that includes 18 core requirements such as having procedures to address potential misconduct, adequate provisions for data preservation, public accessibility of experimental methods, and systems for monitoring performance (96). Each requirement can be translated into verifiable indicators of an institution’s adherence to the quality system.

These examples illustrate innovation in indicators of trustworthiness of research findings. However, development of indicators that are valid, usable, and generalizable is difficult. Substantial investment will be needed to create, test, and adapt indicators for a variety of research circumstances. Evaluation will be essential to determine their validity and reliability, improve indicators over time, and clarify how to use them responsibly and effectively (97). In the end, a healthy science of research assessment will include a diverse set of indicators, with well-understood uses and limitations, a healthy recognition of uncertainty, and a dedication to continuous improvement.

Given the challenges of developing valid, scalable, and appropriate indicators, it is tempting to abandon indicators altogether. However, the absence of indicators is not a solution. Decisions will still be made about what research findings are trustworthy. The absence of valid indicators eliminates meaningful signals and creates a vacuum that leaves decision-makers with little or no guidance. Decision-makers may fall back on idiosyncratic decisions of whether to trust a finding because of who said it, how one heard about it, or whether it just feels right. The research community has a responsibility to pursue research that achieves trustworthiness, and a responsibility to develop indicators to help research consumers assess that trustworthiness.

## Conclusion

The broader literature on trust in science emphasizes multiple criteria that readers and consumers of research bring to their evaluation of research, researchers, and research organizations (75, 98–101). Engaging in research practices that increase the trustworthiness of research findings are preconditions for earning trust. Here, we introduce a systems perspective that focuses on whether the research is accountable, evaluable, well- formulated, has been evaluated, controls for bias, reduces error, and whether the claims are warranted by the evidence. In the process, we focus on the task of developing and improving indicators of trustworthiness.

Trustworthy research practices are the component of earning trust that researchers can control. By adhering to practices that promote the trustworthiness of research findings, researchers contribute to a cumulative body of knowledge that can be relied upon by other researchers, policymakers, practitioners, and the public. In a world of misinformation, ideological campaigns, and motivated reasoning, producing trustworthy research findings may not be sufficient on its own to earn trust, but it is a necessary feature of an enterprise that is relentlessly truth-seeking.

## Supplementary Material

Appendix 01 (PDF)

## Data Availability

There are no data underlying this work.
