# Supplementary material for: A framework for assessing the trustworthiness of scientific research findings
Source: Proc Natl Acad Sci U S A. 2026 Feb 3;123(6):e2536736123. doi: 10.1073/pnas.2536736123 (PMC12890942; doi:10.1073/pnas.2536736123)
Supplement: Supplementary file 1 — Appendix 01 (PDF) [file pnas.2536736123.sapp.pdf]

Supporting information for A Framework for Assessing the Trustworthiness of Research Findings

Illustrations of prior work across several scholarly domains that contribute to assessing trustworthiness of research findings

Lincoln and Guba (1985) proposed four components for assessing trustworthiness of qualitative research: credibility, transferability, dependability, and confirmability. The Belmont Report (1978) offered respect, justice, and beneficence as mid-level principles for promoting the ethics of research with human subjects – a framework ultimately translated into rules and regulations for ethical review of such research. Shadish and colleagues (2002) proposed four types of validity for assessing trustworthiness of findings in experimental and quasi-experimental research: internal validity, external validity, construct validity, and statistical conclusion validity. Nosek and colleagues proposed eight standards for considering transparency and openness as an element of trustworthiness including preregistration and sharing data, materials, and code (Nosek et al., 2015). Bornmann (2011) suggested three key components for assessing trustworthiness of research assessment: reliability, fairness, and predictive validity. Grey and colleagues (2020) offered a checklist with eleven categories to evaluate the integrity of published papers including assessment of statistical reporting, plagiarism, data duplication, and authorship. And, Ciubotariu and Bosch (2022) outlined a framework for promoting responsible scientific communication to research consumers that included objectivity, honesty, openness, accountability, fairness, and stewardship.

Table S1 presents the Trustworthiness Framework for Assessing Research Findings with examples of indicators for each component at each level of the system.

Table S1. *The Trustworthiness Framework for Assessing Research Findings with examples of indicators for each component at each level of the system.*

Levels	**Organizational**	**Researcher**	**Features of**

Components	contributions to trustworthiness of research findings	contributions to trustworthiness of research findings	research findings that promote trustworthiness
**Accountable**: Are the researchers accountable to being trustworthy?	IRB/IACUC/DSMB<br>Research integrity policy<br>COI policy<br>Incentives promoting trustworthy research<br>Compliance monitoring<br>Appropriate staffing to support policies	RCR training of all researchers including trainees<br>Maintaining research records<br>COI Annual Disclosure and management	IRB/IACUC Approval<br>COI Statement<br>Positionality Statement<br>Funding Disclosure<br>Crediting contributors
**Evaluable**: Can the research be assessed?	TOP Policy<br>Transparent review<br>Public Access<br>Maintains or supports repositories	Public website<br>Sharing of research program and content	Shared plans<br>Shared data<br>Shared materials<br>Shared code<br>Shared outcomes
**Evaluated**: Has the research been assessed?	Peer review<br>Promoting replication<br>Personnel assessment	Participation in scholarly societies<br>Engagement in scholarly discourse<br>Solicits and responds to critique	Peer Reviewed<br>Reproduced<br>Robustness<br>Replicated
**Well-formulated**: Does the research take into account relevant knowledge and perspectives?	Supports inclusion and consultation of stakeholders<br>Promotes intellectual diversity<br>Provides tools for accumulating knowledge	Developing skills<br>Relevant expertise<br>Inclusive of perspectives<br>Stakeholder consultation	Literature reviewed and analyzed<br>Theory-informed<br>Representative of and generalizable to relevant population
**Controls bias**: Was the research conducted with accuracy and validity?	Instrumentation investment<br>Providing solutions for identifying bias and verifying/validating claims and measures<br>Skills training	Methods training & competence<br>Using best available methods<br>Solicits critique of methods	Validated measures<br>Blinding<br>Randomization<br>Addressing confounds<br>Preregistration
**Reduces error**: Was the research	Adequate funding for conduct of research	Adequate resources for investigations	Sensitive and specific measurement

conducted with precision and reliability?	Providing solutions for aggregating evidence<br>Skills training	Competent use of analytic and statistical methods	Sample size<br>Reliable measures<br>Power analysis
**Well-calibrated**: Do the claims match the evidence?	Disincentives for spin<br>Mechanisms for critique and self-correction	Seeks counterevidence<br>Exercises care in formulating and presenting claims	Identified uncertainty<br>Stated limitations<br>Identified alternative explanations<br>Identified constraints on generality

Note: IRB = Institutional Review Board; IACUC = Institutional Animal Care and Use Committee; DSMB = Data and Safety Monitoring Board; RCR = Responsible Conduct of Research; COI = Conflict of Interest; TOP = Transparency and Openness Promotion Guidelines (Nosek et al., 2015)

Table S2. *Indicators of the trustworthiness of research findings can be assessed on a variety of dimensions to determine their quality, usefulness, and generalizability.*

Dimensions of Assessment	Explanation	Example A	Example B
Done **versus** ***done well***	The indicator measures whether the action was performed or not versus the indicator assesses the quality of performing the action.	Authors reporting that they randomized the experiment reflects whether the action was performed, not the quality of randomization.	An assessment including whether the dataset is findable, accessible, interoperable, and reusable (FAIR) is determining whether the indicator, shared data, is done well.
Direct **versus** ***proxy***	The indicator assesses the concept itself versus associates of the concept.	Assessing the statistical reliability of measures used to produce a finding is a relatively direct assessment of the findings.	Assessment of an institution's policies for accountability is a relatively indirect assessment of the trustworthiness of research findings as those policies create the conditions for trustworthy findings to be produced.
Self-certification **versus** ***independent verification***	Performance on the indicator is assessed by the actor(s) themselves versus by an independent source.	Authors reporting that they blinded the experiment condition is self-certifiying by the authors that they did it.	An assessment of whether another research group could replicate a finding reflects independent verification of an indicator of replicability.
Occasional **versus** ***continuous***	The indicator is measured once or updated occasionally versus is updated on an ongoing basis.	The indicator, received responsible conduct of research training, would typically be assessed once or occasionally.	Number of publications can be monitored continuously.
Sample **versus** ***census*** **coverage**	The indicator is based on a sample of the occasions that the actions could be performed versus over the entire corpus of occasions.	An indicator of generalizability of a finding based on the published literature could be a sample of evidence due to publication bias ignoring negative and null results.	A meta-analysis assessing the use of validated measures across all preregistered investigations of a finding provides census coverage of registered evidence.

Unstructured* versus *rule-based	The indicator is based on an unstructured or indeterminant assessment process versus a defined rubric or set of rules.	The standard peer review process of papers submitted to journals is unstructured with minimal guidance to reviewers on what and how to review.	Use of the CRediT framework is a structured indicator of contribution and accountability to research.
Human* versus *automated	The indicator is based on human judgment versus the result of an automated process.	Peer assessments, such as judgment of effectiveness of assessing stakeholder interests in the research, is based on human judgment.	Machine learning extraction of evidence for use of reporting guidelines in papers is an automated indicator.
Qualitative* versus *quantitative	The indicator is descriptive versus numerical.	Peer assessment of the quality of a literature review for a paper is usually qualitative.	The indicator, sample size of an experiment, is quantitative.
Thin* versus *thick	Assessment of the indicator draws on a narrow representation of the construct of interest versus a broad representation.	Measuring accountability of the research as exclusively disclosure of conflicts of interest is a thin assessment of accountability.	The indicator, received tenure at an academic institution, is usually the product of a thick assessment of the researchers' scholarly record.
Domain-specific* versus *domain-general	The indicator is applicable to a specific method or topic versus applicable to a wide range of methods and topics.	The indicator, a priori power analysis performed, is specific to methodologies for which power analysis is relevant.	The indicator, peer assessment of researcher engagement in scholarly discourse, can be applied across methods and topics.
Opaque* versus *transparent	How assessment of the indicator is done is unknown or unknowable versus available and understandable to an observer.	An indicator from a black box machine learning assessment of the quality of a paper is opaque as the basis of assessment is unknown.	Publishing peer reviews and editorial letters at a journal provides transparency for the decision to publish a paper and its findings.
Open* versus *proprietary	The assessment is openly available for use and reuse versus owned or controlled by a specific entity.	An openly licensed rubric for evaluating presence of confounds can be used and reused by anyone.	Journal impact factor, as implemented by Clarivate Analytics, is a proprietary measure.
Static* versus *dynamic	The assessment is permanent versus capable of evolving over time.	The indicator, has a PhD, is static once it is marked as true.	The indicator, number of citations of a paper, is dynamic as it accumulates over time.

Retrospective* versus *contemporaneous	The indicator reflects past actions that may or may not correspond to the present versus reflecting the here-and-now.	The indicator, published following peer review at a journal, is retrospective as there is no revisiting the assessment based on contemporaneous knowledge.	A researcher's h-index on Google Scholar is a contemporaneous indicator of their citation impact.

Use cases for a trustworthiness framework and associated indicators

The *Trustworthiness Framework for Assessing Research Findings* can be useful for a variety of purposes in assessing, promoting, and using research findings. The framework illustrates that there are many components of trustworthiness that do not have readily available indicators for assessing whether the actions occurred, or to assess the quality of those actions. The framework can be used to identify and address gaps in assessment with innovations in measurement and development of new indicators. It will be helpful to establish a research field that develops and evaluates indicators to improve coverage of the components of trustworthiness, and the usability of the indicators. Also, research communities can use the framework to review where their community norms and practices can effectively assess trustworthiness, and where more investment and attention is needed.

Researchers might use the components and indicators of trustworthiness in planning, conducting, and evaluating their own research. Labs might define new norms for how to meet as many of the indicators of trustworthiness as possible as a standard operating procedure. Research teams might adapt indicators to be appropriate for the specific purpose in their research context.

Researchers might also use the components and indicators in evaluating research by others. As reviewers, the framework offers guidance on what features to assess, critique, and make recommendations about to enhance the credibility of papers and findings. In planning research agendas, the framework might provide guidance on which areas of relevant scholarship are credible enough to use in building a new program of research.

Research institutions might use the framework to assess the quality and gaps in their services supporting research. Universities might identify opportunities to provide support or training services that would increase trustworthiness of work at the institution. Funders might recognize opportunities to support development and validation of new indicators that are particularly relevant to the domains and methods that align with their funding priorities. And, journals might evolve their policies and procedures so that more components of the framework are incorporated into the peer review and publication process.

Research institutions might also use the framework to improve their assessment of researchers. At universities, hiring, promotion, and tenure policies might incorporate more direct indicators of research trustworthiness rather than relying heavily on indicators such as number of publications and journal reputation. And, among funders, criteria for selecting proposals and expectations for productivity might incorporate indicators that support producing trustworthy findings.

Eventually, a maturing constellation of indicators that address the diversity of research methods, epistemologies, and domains could provide a more nuanced and comprehensive evidence base for assessing the trustworthiness of research. This would support multiple uses. For example, incorporating direct evidence of trustworthiness into communication with journalists,

policymakers, and the wider public would help those stakeholders make more informed and accurate decisions based on assessment of the available research.
